# Association of Ego Defense Mechanisms with Academic Performance, Anxiety and Depression in Medical Students: A Mixed Methods Study

**DOI:** 10.7759/cureus.337

**Published:** 2015-09-30

**Authors:** Ahmed Waqas, Abdul Rehman, Aamenah Malik, Umer Muhammad, Sarah Khan, Nadia Mahmood

**Affiliations:** 1 Final year MBBS Student, CMH Lahore Medical College and Institute of Dentistry, Shami Road, Lahore Cantt.; 2 Medical Student, Allama Iqbal Medical College, Lahore, Pakistan; 3 Department of Biochemistry, CMH Lahore Medical College and Institute of Dentistry, Shami Road, Lahore Cantt.; 4 Department of Psychiatry, Combined Military Hospital, Lahore, Pakistan; 5 Department of Pathology, CMH Lahore Medical College and Institute of Dentistry, Shami Road, Lahore Cantt.

**Keywords:** ego defense mechanisms, medical students, anxiety, depression, academic performance

## Abstract

Background: Ego defense mechanisms are unconscious psychological processes that help an individual to prevent anxiety when exposed to a stressful situation. These mechanisms are important in psychiatric practice to assess an individual’s personality dynamics, psychopathologies, and modes of coping with stressful situations, and hence, to design appropriate individualized treatment. Our study delineates the relationship of ego defense mechanisms with anxiety, depression, and academic performance of Pakistani medical students.

Methods: This cross-sectional study was done at CMH Lahore Medical College and Fatima Memorial Hospital Medical and Dental College, both in Lahore, Pakistan, from December 1, 2014 to January 15, 2015. Convenience sampling was used and only students who agreed to take part in this study were included. The questionnaire consisted of three sections: 1) Demographics, documenting demographic data and academic scores on participants’ most recent exams; 2) Hospital Anxiety and Depression Scale (HADS); and 3) Defense Style Questionnaire-40 (DSQ-40). The data were analyzed with SPSS v. 20. Mean scores and frequencies were calculated for demographic variables and ego defense mechanisms. Bivariate correlations, one-way ANOVA, and multiple linear regression were used to identify associations between academic scores, demographics, ego defense mechanisms, anxiety, and depression.

Results: A total of 409 medical students participated, of whom 286 (70%) were females and 123 (30%) were males. Mean percentage score on the most recent exams was 75.6% in medical students. Bivariate correlation revealed a direct association between mature and neurotic ego defense mechanisms and academic performance, and an indirect association between immature mechanisms and academic performance. One-way ANOVA showed that moderate levels of anxiety (P < .05) and low levels of depression (P < .05) were associated with higher academic performance.

Conclusion: There was a significant association between academic performance and ego defense mechanisms, anxiety, and depression levels in our sample of Pakistani medical students.

## Introduction

Ego defense mechanisms are unconscious psychological processes that help an individual cope with anxiety resulting from a stressful internal or external environment. Defense mechanisms find their origin in Freud’s structural theory of the mind, which divides the human mind into three entities: internal drive (id), ego, and superego [1]. The id comprises an individual’s unconscious instincts, such as libido. These basic unconscious desires are checked by the reins of the ego, the executive organ of the psyche that, in contact with reality, modulates desires of the id through the use of defense mechanisms. Ego, in turn, is under the influence of superego, which underlies the desires for perfection, ideals, and spiritual goals. This interaction of ego and superego gives rise to morality, guilt, and conscience [2]. The necessity of defense mechanisms arises when the demands of the animalistic id contradict with those of the idealistic superego. In order to maintain mental homeostasis and protect the conscious mind from the effects of such conflicts, the ego makes use of various defense mechanisms. These mechanisms are thought to be of paramount importance, not only in maintaining mental stability in normal individuals but also in determining psychopathologies in psychiatric patients.

Freud first described ego defense mechanisms in 1894 and suggested a possible association between psychiatric illnesses and these psychological processes [3-4]. Later, Anna Freud strengthened the theory based on her clinical experience; Vaillant described the hierarchical nature of these defenses and grouped them on a continuum of ego maturity from immature to neurotic to mature [1, 5]. Mature defense mechanisms are associated with adaptive functioning, in contrast to immature defenses and neurotic defense mechanisms, which – despite being pathological and associated with high anxiety levels – represent an individual’s effort to maintain psychological homeostasis in response to a stressful environment [6-7].

Like many other tenets of the Freud’s psychoanalytic theory, the validity of defense mechanisms and the usefulness of their measurement have been called into question. However, it is argued that defenses can be inferred accurately from conscious derivatives as reported by the subjects [8]. Defense mechanisms so measured can then be used as a framework to elucidate personality characteristics, psychosocial functioning, temperament, stressful life events, mental health, physical health, and psychopathologies of individuals [8-13]. It has also been suggested that mental status or psychiatric evaluation of an individual should be accompanied with identification of the subject’s dominant defense mechanisms, and sometimes a return to the Freudian defense mechanisms is also required [14-15]. Ego defenses were also included in the DSM-IV among the axes suggested for further study [16]. Thus, it is evident from a vast body of literature on the subject that defense mechanisms and their measurement remain relevant in both theory and practice of modern psychiatry.

The field of medicine is inherently a very stressful field of education, exposing medical students to a plethora of academic and psychosocial stressors [17]. Medical students also have unique personality characteristics. They are often considered high achievers, perfectionists, and possessing more Type A traits than their counterparts in other academic fields [18]. The demanding professional commitments of training at a medical school have profound effects on their personalities and psychological health [19]. Thus, it is hardly a surprise that there is a vast body of literature highlighting greater psychological morbidity among medical students. For instance, several studies have documented a very high incidence of anxiety, depression, stress, and sleeping difficulties among medical students [20-21].

The occurrence of psychological problems in medical students, such as anxiety and depression, may be associated with impairments in their ego defense mechanisms, as suggested by several studies in general population that have linked impaired defense mechanisms with depression, dysthymia, mania, panic disorder, and personality disorders [22-26]. Although ego defense mechanisms and their association with psychiatric problems, like anxiety and depression, have been studied extensively in general population, there is a dearth of studies elucidating the ego defense mechanisms employed by medical students and their relation to high level of psychiatric problems commonly observed among medical students.

To our knowledge, only one study has investigated the defense mechanisms of medical students in Pakistan according to which the most commonly employed ego defenses by medical students were rationalization, anticipation, and undoing while the defenses of devaluation, denial, and displacement were least common [27]. However, the study by Parekh, et al. was limited to the prevalence of defense mechanisms and did not assess associations of ego defenses with anxiety, depression, or psychosocial functioning in medical students [27]. In a similar study using the Defense Style Questionnaire-40 (DSQ-40), La Cour reported a higher use of pseudo-altruism, dissociation, denial, sublimation, and suppression in medical students than high school students. He also highlighted the pattern of defense mechanisms among medical students and speculated their potential use in dealing with both academic and psychosocial stressors [28]. However, La Cour again did not use any comparator scales to strengthen his speculations about the potential applications of ego defenses by medical students [28].

Thus, existing studies on defense mechanisms of medical students have ignored the implications of these defenses on their mental health and academic performance. Given the increasing incidence of psychiatric problems among medical professionals and the importance of the medical profession for society, it is necessary to describe these implications in greater detail so that strategies can be employed to promote the psychologically healthy and performance-enhancing defense mechanisms and discourage the opposite mechanisms. These mental health promoting strategies among medical students are indeed a need of the hour as is clear from the literature, which shows that mature defense mechanisms are associated with better functioning, problem-solving, and resolution coping mechanisms, whereas immature mechanisms are associated with escape and evasion coping mechanisms, and thus, might lead to better academic achievements [29-30]. We attempt to bridge this gap in scientific knowledge in the hope that it will help increase the number of high-performing and psychologically healthy doctors.

Thus, our study attempts to explore the ego defense mechanisms in the medical students of Pakistan and has been designed with the following objectives:

1) To evaluate the prevalence of ego defense mechanisms used by medical students.

2) To analyze the association of ego defense mechanisms with anxiety in medical students.

3) To analyze the association of ego defense mechanisms with depression in medical students.

4) To analyze the association of ego defense mechanisms with medical students’ academic performance in their annual examinations.

5) To analyze the association of academic performance of medical students with anxiety and depression.

In accordance with the objectives of the present study, we tested the following hypotheses:

H1: Use of mature defenses rather than neurotic or immature defenses is more common in medical students.

H2: Higher anxiety levels are associated with higher use of neurotic and immature ego defenses and lesser use of mature ego defenses.

H3: Higher depression levels are associated with the higher use of neurotic and immature ego defenses and the lesser use of mature ego defenses.

H4: Higher academic achievement is associated with the higher use of mature ego defenses and the lesser use of neurotic and immature defenses.

H5: Higher academic achievement is associated with low anxiety levels and low depression levels.

## Materials and methods

### Study design

This mixed methods study was undertaken at the CMH Lahore Medical College (LMC) and Institute of Dentistry and the Fatima Memorial College of Medicine and Dentistry, both in Lahore, Pakistan. Ethical approval for this study was obtained from the CMH LMC Research Ethics Committee. No IRB numbers are allotted by the mentioned research ethics review committee. Informed consent was obtained from all participants.

### Study population

CMH Lahore Medical College (LMC) and Institute of Dentistry and the Fatima Memorial College of Medicine and Dentistry, both in Lahore, Pakistan, are privately financed medical colleges affiliated with the University of Health Sciences (UHS), Lahore, Pakistan. The UHS offers a five-year medical degree program (bachelor degree in medicine and a bachelor degree in surgery, MBBS) divided into two pre-clinical years and three clinical years. The affiliated colleges follow the curriculum and teaching guidelines set by the UHS. The academic year culminates with an annual examination held by the UHS, which is mandatory to pass to get promoted to the next year.

### Pilot survey

A pilot survey was conducted among 30 medical students to ensure that the questionnaire could be understood by them with ease. Their feedback was requested to further improve the questionnaire. This was not included in the final analysis.

### Sample Size

Generally, in social sciences, a sample-size calculation is based on the variability in the sample and anticipated effect sizes. Both of these were unknown before starting the study, and no power estimates were available for multiple regression analysis employed in the study. According to Vanvoorhis, et al., for regression equations employing six or more predictors, a minimum of 10 participants per predictor variable should be included in the study [31].

We used a random sampling approach to data collection and a total of 500 self-administered questionnaires were distributed among medical students, selected through a computer software, to ensure an adequate percentage response rate. Students were given a brief introduction to the background of this study and the questionnaire used. The students were then given a dedicated time period (25 minutes) during their class lecture to complete the questionnaire. Only students who had volunteered to take part in this study were included. Written informed consent was obtained from all participants, and they were assured anonymity and that only group-level findings would be reported.

### Questionnaire

The questionnaire consisted of three sections: 1) a section recording demographics and percentage grades obtained on their annual examination, 2) the Hospital Anxiety and Depression scale (HADS), and 3) the Defense Style Questionnaire-40 (DSQ-40). Grades obtained in their annual examinations were confirmed through academic records of each respondent during the collection of questionnaires by data collectors.

The HADS is a 14-item self-administered questionnaire and one of the most extensively used questionnaires in Pakistan to assesses the respondents’ levels of anxiety and depression in both the hospital setting and the general population [32]. It was cross-culturally validated in a sample of Pakistani medical students [32]. It comprises two subscales, each with a score ranging from 0 to 21. This score is classified as no anxiety/depression (0-7), borderline anxious/depressed (8-10), and severely anxious/depressed (11-21). A systematic analysis of this instrument evaluated and verified its criterion validity and cross-cultural validity in Pakistan [33].

The DSQ-40 is one of the most widely used psychometric instruments for assessing ego defense mechanisms used by respondents [34]. It has demonstrated good construct and content validity by discriminating between different psychiatric populations in various studies and adequate reliability statistics with test-retest reliability (.66) and high inter-item correlations (.78) [13, 34-35]. It is one of the most extensively used self-report measures to assess ego defense mechanisms in various countries, such as Pakistan, Iran, Finland, Canada, Brazil, Japan, and Denmark, both in psychiatric patients, medical students, and the general public of varying ages [27-28, 36-40].

This questionnaire was chosen because it is short, convenient to understand, and also has enough items to distinguish different defense styles [34]. Previous studies reporting ego defenses in medical students in Pakistan have also employed DSQ-40 rather than the longer versions, such as DSQ-67 (67 items) and Bond’s DSQ (88 items) [27]. The DSQ-40 has also demonstrated similar results to longer versions, such as DSQ-67 [34].

It broadly categorizes these mechanisms into three hierarchies: 1) mature, 2) neurotic, and 3) immature defense mechanisms, similar to Vaillant’s hierarchy of ego defense mechanisms. The defense mechanisms have further been classified by Andrews into: (a) four mature: sublimation, humor, anticipation, and suppression; (b) four neurotic: undoing, pseudo-altruism, idealization, and reaction formation; and (c) twelve immature: projection, passive aggression, acting out, isolation, devaluation, autistic fantasy, denial, displacement, dissociation, splitting, rationalization, and somatization. Each type is covered by 2 items in the DSQ-40 [34]. The average scores for the two items are used to determine individual defense mechanisms. The average scores for specific ego defense mechanisms are then grouped into mature, neurotic, and immature categories for the purpose of data analysis.

Most of the studies employ the scoring of DSQ-40 as mentioned above, but Ruuttu, et al. suggested that a score representing the overall functioning of ego defenses should be employed rather than reporting hierarchies of ego defenses [37]. However, for the sake of analysis, overall functioning is not being reported in the present study.

### Focused group and individual interviews:

Focused groups and individual interviews were conducted on a subsample of the respondents. The interviews were closed via the criteria of saturation, resulting in a total of 15 interviewees. These interviews were semi-structured with open-ended questions. The interview questions comprised several academic and psychosocial stressors, and the responses of the candidates were recorded. Names of the respondents were changed, and they were ensured anonymity. Their responses were then transcribed, themes were developed, and then analyzed by an experienced psychiatrist in the perspective of psychodynamics.

### Data analysis

All data were analyzed with SPSS v.21 software. Frequencies were calculated for demographic characteristics, levels of anxiety, and levels of depression as measured by the HADS. In addition, mean scores (standard deviation) were calculated for anxiety and depression subscales of HADS as well as for the mature, neurotic, and immature subscales of DSQ-40.

A bivariate correlation (Pearson correlation) was used to identify associations between defense styles and i) percentage of marks obtained in the annual examination, ii) anxiety subscale scores, and iii) scores on a depression subscale.

Multiple regression analysis (backward method) was conducted separately to identify significant predictors of i) percentage of marks obtained in the annual examination, ii) mean scores on an anxiety subscale, and iii) mean scores on a depression subscale. Age, the gender of medical students, and mean scores on individual ego defenses mechanisms were entered as predictors in the multiple regression analysis. Twenty-three (23) predictors, including age, gender, year of education, and twenty defense mechanisms as assessed by the DSQ-40, were added in each regression model satisfying the mentioned minimum sample size criteria as defined by Van Voorhis, et al. [31].

One-way analysis of variance (ANOVA) and posthoc least statistical difference test (LSD) were used to analyze associations between levels of anxiety, depression, and percentage of marks obtained by medical students in annual examinations.

Normality of the quantitative data was checked with histograms and QQ plots. Durbin-Watson diagnostics, collinearity diagnostics, the values of variance inflation factor (VIF), tolerance statistic (TOL), and influential points were checked to ensure that the data did not violate the assumptions of multiple regression analysis.

## Results

The total percentage response rate was 81.8% (409/500). The mean age of the respondents was 19.9 (1.33) years. Their mean HADS scores were 9.5 (3.59) on the anxiety subscale and 5.8 (3.12) on the depression subscale. Mean scores on the DSQ-40 were 5.6 (1.19) for mature, 5.8 (1.20) for neurotic, and 5.0 (.91) for immature ego defense mechanisms. Participants’ mean percentage score on their annual medical school examinations was 75.6% (9.12%). According to the HADS, out of 409 medical students, 132 (32.3%) were borderline anxious, 151 (37%) were severely anxious, 83 (20.3%) were borderline depressed, and 35 (8.6%) were severely depressed.

### Mean scores on ego defenses:

Most commonly employed ego defenses by medical students were rationalization, anticipation, pseudo-altruism, undoing, and humor, whereas the least commonly employed defense mechanisms were devaluation, denial, and dissociation (Table 1).

**Table 1:** Mean scores and standard deviations on ego defense mechanisms

Ego Defense Mechanism	Mean	Standard Deviation
Sublimation	5.27	1.82
Humor	5.69	1.91
Anticipation	6.10	1.75
Suppression	5.32	1.92
Undoing	5.97	1.76
Pseudo altruism	6.13	1.70
Idealization	5.71	2.10
Reaction formation	5.38	1.97
Projection	4.69	1.78
Passive aggression	4.69	1.83
Acting out	5.57	2.01
Isolation	5.48	2.13
Devaluation	4.01	1.78
Autistic fantasy	5.36	2.19
Denial	4.14	1.93
Displacement	4.62	1.98
Disassociation	4.46	1.96
Splitting	5.28	1.83
Rationalization	6.14	1.79
Somatization	5.26	2.13

### Gender and study year:

According to t-tests for independent samples, female students had higher scores for neurotic defense mechanisms (mean difference .29, p < .05), whereas no significant difference were reported on mature (p = .62) and immature defense mechanisms (p = .45). Students enrolled in the preclinical years of the degree program (mean difference .37, p < .01) had higher scores for neurotic defense mechanisms than their counterparts enrolled in later (clinical) years while there were no significant associations between study year and mature (p =.24) and immature defense mechanisms (p = .27).

### Defense styles:

Bivariate correlation revealed a direct association between mature and neurotic ego defense mechanisms and academic performance, and an indirect association between immature mechanisms and academic performance (Table 2).

**Table 2:** Bivariate correlations between hierarchies of ego defenses, anxiety, depression, and marks in annual exams * denotes P < .05, ** denotes P < .01, *** denotes P < .001, 1 denotes P = .05

	Depression	Anxiety	Marks Percentage
Mature	-.209***	-.155**	.123*
Neurotic	.024	.188***	.112*
Immature	.115*	160**	-.096

### Predictors of academic performance:

According to multiple regression analysis, ego defenses like humor, pseudo-altruism, and rationalization were positive predictors of academic performance, whereas the age of the respondents, projection, and displacement were negatively associated with it (Table 3).

**Table 3:** Multiple regression analysis (backward) for academic performance Adjusted R2= .37, ANOVA P < .001

Predictors	B	Std. Error B	Beta	P-value
(Constant)	147.059	5.907		.000
Age	-3.691	.276	-.538	.000
Humor	.339	.201	.071	.093
Pseudo altruism	.410	.225	.076	.069
Projection	-.488	.214	-.095	.023
Displacement	-.545	.195	-.118	.006
Rationalization	.368	.221	.072	.097

### Association of academic performance with anxiety and depression:

One-way ANOVA revealed significant differences between anxiety levels (F = 4.7, df= 2, P = .01) and depression levels (F = 4.9, df = 2, P < .01) on mean percentage of marks obtained in their annual examinations.

Post-hoc LSD tests revealed that borderline anxious students scored better on their annual examination than students who were not anxious (mean difference: 2.4, S.E. 1.1, and P < .05) or severely anxious (mean difference: 3.2, S.E. 1.1, and P < .01). Less depressed students scored better on their annual examination than borderline (mean difference: 2.7, S.E. 1.1, and P < .05) or severely depressed students (mean difference: 4, S.E. 1.6, and P < .05). However, the difference in mean scores between moderately and severely depressed students was not significant (mean difference = 1.3, S.E. = 1.8, P > .05).

### Predictors of anxiety in medical students:

According to multiple regression analysis, female gender, idealization, reaction formation, autistic fantasy, displacement, splitting, and somatization were associated positively with anxiety scores, whereas suppression, denial, dissociation, and rationalization were associated negatively with it. (Table 4)

**Table 4:** Multiple regression analysis for anxiety scores R2= .357, ANOVA P < .001

Variables	B	Std. error B	Beta	P-value
(Constant)	7.020	1.094		.000
Gender	.745	.338	.098	.028
Suppression	-.303	.087	-.158	.001
Idealization	.178	.084	.101	.034
Reaction formation	.203	.085	.108	.017
Autistic fantasy	.283	.077	.168	.000
Denial	-.240	.093	-.125	.010
Displacement	.180	.086	.096	.036
Dissociation	-.213	.093	-.113	.022
Splitting	.189	.095	.093	.047
Rationalization	-.365	.096	-.177	.000
Somatization	.303	.079	.174	.000

### Predictors of depression in medical students:

According to multiple regression analysis, depression scores were positively associated with age, idealization, passive aggression, isolation, devaluation, and somatization, while suppression, humor, dissociation, and rationalization were negatively associated with it (Table 5).

**Table 5:** Multiple regression analysis for depression scores

Predictors	B	Std. error B	Beta	P-value
(Constant)	.212	2.333		.928
Age	.283	.108	.123	.009
Suppression	-.249	.079	-.156	.002
Humor	-.192	.080	-.119	.017
Idealization	.186	.071	.127	.009
Passive aggression	.169	.084	.101	.046
Isolation	.164	.071	.113	.022
Devaluation	.212	.085	.123	.013
Dissociation	-.175	.079	-.111	.027
Rationalization	-.202	.087	-.118	.021
Somatization	.149	.070	.103	.035

## Discussion

The participants in our study scored higher on neurotic defense style than on mature or immature styles. This result is in agreement with Parekh, et al., who reported that the neurotic defense style were more prevalent than either mature or immature defense styles among medical students in Karachi [27]. Among individual defense mechanisms, we found rationalization, pseudo-altruism, and anticipation to be the most commonly employed defense mechanisms among the medical students. These results are in agreement with Parekh, et al. and La Cour who found a high prevalence of these defense mechanisms among the medical students [27-28].

Our study showed a high prevalence of anxiety and depression among medical students, which is in agreement with earlier research on the subject. For example, a systematic review of anxiety, depression, and burnout among medical students in the USA and Canada found that the prevalence of these problems was clearly higher in medical students than in the general population [41]. Studies in Pakistan have yielded similar results, with a prevalence of anxiety and depression among medical students of 43.7% in Multan and 70% in Karachi [42-43]. The exact causes of greater anxiety and depression among medical students have yet to be clearly determined but are probably related to academic stress as demonstrated by Shaw, et al. and Sreeramareddy, et al. [18, 20]. Studies in Pakistan have also identified academic stressors as the main contributors to anxiety and depression in medical students [17, 21]. However, it is not clear whether medical education is more stressful than other forms of higher education [44]. Further research is needed to delineate the exact causes of anxiety and depression among medical students.

Female students were found to have higher levels of anxiety and a higher score on neurotic defense style than their male counterparts did. Higher anxiety among females is well documented in both the general population and among students [45-46]. This finding may be explainable in part by biological, genetic, and social differences between the two genders [45].

Female students are generally more studious than males and tend to be more aware of their own drawbacks and deficiencies, which results in not only in greater stress but also better academic performance [46]. The finding that higher scores on neurotic defense style were more frequent among female students is supported by several studies, including a study of medical students in Pakistan and a study of psychology undergraduates in the USA [27, 47]. La Cour explained that more prevalent neurotic defense styles in female medical students may be due to the fact that they internalize what they learn in class [28]. This is further elaborated by Diehl et al, who concluded that women tend to use more internalizing defense mechanisms than men [48]. This internalization may explain our finding of more frequent neurotic defense mechanisms in our subsample of women compared to men.

Students enrolled in preclinical years of the medical degree program scored higher on neurotic defense style than students in clinical years. This result is consistent with Parekh, et al., who found that students enrolled in preclinical years of their degree scored higher on neurotic and immature defense styles [27]. This may be explained by the fact that students in clinical training become accustomed to the increased stress over the years, and thus develop more effective coping skills compared to those in preclinical training who are new to medicine and are adapting to the stressful demands of medical education. More research is advisable to explore the neurotic defense style among medical students.

Our study found a significant association between academic performance and levels of anxiety and depression. Students with moderate anxiety scored better than those with low or severe anxiety, and students with lower scores on depression subscale scored better than those with moderate or severe depression. Chapell, et al., who found that among 5,551 undergraduate and graduate students in the USA, students with higher test anxiety (particularly females) had a higher grade point average than their counterparts with lower test anxiety [49], supports our findings. However, our findings contradict, in part, those of Cassady, et al., who compared groups of students with low, average, and high anxiety and found that those with low anxiety performed better than the other two groups [50]. Our result finds its explanation in the Yerkes Dodson’s curve according to which a moderate level of anxiety/arousal increases performance while both low and high levels impair performance [51]. However, the same can’t be said for depression because even a moderate level of depression, with its associated feelings of hopelessness and lack of motivation, is predictably associated with poor academic performance. For instance, Hysenbegasi, et al. found that both diagnosed and self-reported depression were associated with decreased academic productivity in university students [52].

Looking at the hierarchies of defense mechanisms, we see many interesting findings. Despite some individual variations (which are discussed below), taken as a whole, mature defense style was associated with better academic performance, lower anxiety, and lower depression while immature and neurotic defense styles were associated with relatively lower academic performance, higher anxiety, and higher depression levels. These findings are in agreement with a vast body of literature. For instance, Grebot, et al. found that mature mechanisms are associated with problem-solving coping mechanisms, whereas immature mechanisms are associated with escape and evasion coping mechanisms [29]. The type of coping mechanisms employed is then responsible for better academic performance in students using mature defense mechanisms and poor performance in those who employ immature mechanisms as shown by MacCann, et al. [30]. Several statements made by the medical students of our study suggest that mature defense mechanisms drive the students employing these mechanisms to work harder and perform better in their exams. For instance, one student reported: “I don’t study much because my parents aren’t really concerned with it. I only study because I am afraid that I would fail my annual exams” (anticipation), and another said: “My father had a massive stroke and became bedridden 2 years ago. It changed my life completely. I could have used it as an excuse to fail tests. Instead, I worked harder and became a better person to meet up to his expectations” (sublimation). Similarly, the findings of low depression and anxiety with the use of mature mechanisms and high depression and anxiety with immature defense mechanisms are supported by several studies, including those by Spinhoven and Kooiman, Blaya, et al., Carvalho, et al., and Sarisoy [22, 53-55]. Bowins, in a review of defense mechanisms, explains the reason for this finding [56]. He says that mature defense mechanisms promote mental health since they allow an individual to view his environment in a positive, albeit slightly distorted, fashion boosting his self-esteem and protecting him against depression and anxiety. The following statement by a student hints towards how mature mechanisms protect from anxiety and depression: “I have so many exams these days. I get extremely burnt out and start having negative thoughts. I cope with it by painting, listening to music, or sports” (sublimation and suppression). In contrast, immature defense mechanisms, while having an adaptive value, are associated with extreme distortions and are more likely to be associated with psychiatric manifestations, including anxiety and depression. Thus, we advise the incorporation of programs in medical education to identify the defense mechanisms of medical students and promote adaptive mechanisms by strategies designed for this purpose. For instance, behavioral therapy and meditation have been shown to increase the use of adaptive (mature) defense mechanisms [57-58]. This, in turn, would improve both the performance and mental health of medical students, as proven by our study.

However, a few defense mechanisms had unexpected associations. For instance, a greater use of rationalization was associated not only with low anxiety and depression but also with better academic performance. It is well known that rationalization is one of the major defense mechanisms employed by medical students, as found by Parekh, et al. and La Cour [27-28]. La Cour explained that the greater use of rationalization allows medical students to adapt to the grim realities they face in hospitals, e.g., disease and death. In our sample, many medical students utilized rationalization to adapt to many academic stresses as well, which is suggested by several statements. For instance, one student reported: “I had excellent scores in A levels. I couldn’t get enrolled in a public medical college because I scored less in equivalency certificate. Now I feel that whatever had happened might be for good.” Other students rationalized cheating behaviors in their exams, for instance, “If I don’t prepare for an exam, I cheat. Because everyone else is doing it”. When seen in this light, our paradoxical finding makes sense. Thus, medical students who use greater rationalization are better able to cope with the psychological and academic challenges that medical profession presents them; as a result, they experience less anxiety and depression and perform better on exams than students who do not use rationalization.

Dissociation and denial also showed surprising associations in our study. Despite the fact that both of them are classified as immature, we found that in medical students, higher scores on dissociation was protective against both depression and anxiety while greater use of denial was protective against depression. Both of these defenses have traditionally been considered to be associated with psychopathology, particularly, psychosis [59-60]. However, recently several authors have realized the adaptive importance of these defenses. For instance, in his review of defense mechanisms, Bowins states that mild forms of dissociation, such as depersonalization or derealization and denial, are not only quite common in general population but are also highly adaptive in acute stress [56]. Subjected suddenly to the stresses of death and suffering in hospitals, medical students resort to these defense mechanisms to maintain psychological health. Several statements of students in our study suggest this. For instance, one of the students reported a mild episode of dissociation after witnessing a patient’s death for the first time: “I experienced my first patient death in the third year of MBBS. It was so devastating for me that I had lost my senses. I was baffled with the reality of nature and kept questioning it. I stayed in this condition for a week.” Another student hinted the use of denial by saying: “I am amazed at how little we are aware of deaths happening around us in wards.” However, while these defense mechanisms may improve the mental health of doctors, at the same time, they may also increase their emotional rigidity and cause emotional detachment from their patients. This may underlie the decreasing empathy towards patients among medical professionals that has been pointed out with much concern in recent years [61]. Informing the students of the negative effects of these defense mechanisms may help them protect themselves from emotional rigidity. This is indeed a need of the hour because a recent study has reported that Pakistani medical students are least patient-centered than students in other countries [21].

Pseudo-altruism was found to be associated with better performance in the annual examination. Vaillant, et al. also described the greater use of altruism and self-sacrifice by physicians but did not view it as maladaptive [62]. Our study, in fact, highlights the adaptive nature of this neurotic defense mechanism, which can be explained by the fact that the medical students employing pseudo-altruism assume more responsibility towards health of their future patients than other medical students. A statement by one of the medical students proves how altruism can promote hard work: “I study hard because patients’ lives depend on me.” This increased the sense of responsibility in students employing altruism drives them to work harder and get better grades in the exams.

### Limitations

The cross-sectional design of this study limits inferences about causality and temporality. Levels of anxiety and depression were assessed with the HADS, so the results are not completely transposable to the clinical criteria used to reach the diagnoses. In addition, the use of a self-administered questionnaire may lead to information bias. Although the DSQ-40 has demonstrated good construct validity and reliability in various studies and cultures, according to Trijsburg, et al., items of the DSQ-40 should rather be represented unidimensionally instead of a three-factor structure entailing mature, neurotic, and immature factors [63]. However, this study has focused on a three-factor construct of the DSQ-40 as suggested by Andrews (13).

## Conclusions

The prevalence of anxiety and depression among medical students in our sample was quite high. Higher academic scores were associated with moderate anxiety levels and low depression levels. Academic performance, anxiety, and depression levels correlated significantly with ego defense mechanisms. Spreading awareness of defense mechanisms among the medical students may enable them to employ more mature defense mechanisms and avoid the negative effects of several immature defense mechanisms, such as dissociation and denial. 
